# Identifying and ranking of CMIP6-global climate models for projected changes in temperature over Indian subcontinent

**DOI:** 10.1038/s41598-024-52275-1

**Published:** 2024-02-06

**Authors:** Abdul Rahman, Sreeja Pekkat

**Affiliations:** Department of Civil Engineering, Indian Institute of Technology Guwahati, Guwahati, 781039 Assam India

**Keywords:** Climate change, Civil engineering

## Abstract

Selecting the best region-specific climate models is a precursor information for quantifying the climate change impact studies on hydraulic/hydrological projects and extreme heat events. A crucial step in lowering GCMs simulation-related uncertainty is identifying skilled GCMs based on their ranking. This research performed a critical assessment of 30 general circulation models (GCMs) from CMIP6 (IPCC’s sixth assessment report) for maximum and minimum temperature over Indian subcontinent. The daily temperature data from 1965 to 2014 were considered to quantify maximum and minimum temperatures using a gridded spatial resolution of 1°. The Nash–Sutcliffe efficiency (NSE), correlation coefficient (CC), Perkins skill score (PSS), normalized root mean square error (NRMSE), and absolute normalized mean bias error (ANMBE) were employed as performance indicators for two different scenarios, S1 and S2. The entropy approach was used to allocate weights to each performance indicator for relative ranking. Individual ranking at each grid was achieved using a multicriteria decision-making technique, VIKOR. The combined ranking was accomplished by integrating group decision-making, average ranking perspective, and cumulative percentage coverage of India. The outcome reveals that for S1 and S2, NRMSE and NSE are the most significant indicators, respectively whereas CC is the least significant indicator in both cases. This study identifies ensemble of KIOST-ESM, MRI-ESM2-0, MIROC6, NESM3, and CanESM5 for maximum temperature and E3SM-1-0, NESM3, CanESM5, GFDL-CM4, INM-CM5-0, and CMCC-ESM2 for minimum temperature.

## Introduction

Temperature and precipitation are the most widely used climatic parameter that unveils the impact of climate change over a region. Alteration in local water availability for irrigation purposes, occurrences of extreme events like droughts and floods, change in temperature patterns and severe heat wave occurrences are some of the common climate change impacts on society^1^. To tackle the above problems and have better infrastructure planning for the future, it is important to predict the impacts of climate change in terms of temperature and/ or precipitation. Global Climate Models (GCMs) are used for projecting future climatic data that can be used for hydro-climatological studies. Several studies worldwide consider climatic variables like maximum and minimum temperature, precipitation, surface mean temperature, and sea surface temperature for simulating GCMs in combination with the observed data^2–11^.

The factors like complex topography of a region, monsoon dynamics with its onset, strength, and duration are influenced by the atmosphere, land, and ocean's complex interaction^12,13^. The natural climate variabilities like El Nino-Southern Oscillation (ENSO) and Indian Ocean Dipole (IOD) often biases GCMs’ maximum and minimum temperatures in different seasons^14,15^. The correlation between surface temperature and precipitation involving 17 CMIP5 GCMs, observed that models performed better in the cold season than in the warm season, and better over the land than over the oceans of the Indian subcontinent^16^. Low-frequency air eddies may alter global and regional climate over decadal periods^17^.

There are various other uncertainties associated with the GCM simulations such as inappropriate parameterization of aerosols, initial and boundary conditions, greenhouse gas emission, systematic model errors, and socio-economic factors making it challenging to use at local and regional scales. The additional uncertainties like random internal climate variability, scenarios of indefinite change due to anthropogenic activities, and physical responses by model equation arising during downscaling to regional scale^2,18^. Also, sometimes a limited number of GCMs are used for climate impact studies due to time and resource constraints. Hence, a detailed evaluation is important for selecting a suitable GCM or ensemble of GCMs (from a pool of available GCMs) before employing them for climate change impact studies.

CMIP6 models are upgraded versions of the models that took part in prior phases of CMIP, in terms of increased spatial resolution, physical parameterizations (such as cloud microphysics), better carbon–nitrogen cycle parameterizations, and better aerosol representation^19^. Nonetheless, there is still a need to analyze the accuracy and dependability of GCMs for modeling historical data in order to determine how well they simulate previous climatic conditions and to make efforts to lessen the uncertainty associated with future climate projections. Ranking of GCMs and suggesting the best ensemble GCMs for certain areas are one of the ways to reduce the uncertainty related to the selection of GCMs. Several studies were conducted for climatic variables including maximum and minimum temperatures, average temperatures, and precipitation. Some of the studies include, a relative ranking of 36 CMIP6 GCMs across Telangana state^2^, 36 CMIP5 GCMs over India^11^, 11 CMIP6 based HighRes MIP over India^5^, and 26 CMIP6 GCMs over the Krishna river basin, India^3^. For the evaluation of GCMs’ ability to simulate historical observations, several simple, effective, and meaningful performance indicators are used^2,10,20^. Some of the performance indicators considered for ranking GCMs are correlation coefficient (CC), normalized root mean square error (NRMSE), and skill score (SS)^11^. Different weights were assigned by using entropy method and the ranking of GCMS were carried out by compromise programming method^11^.

Skill score-based indicator is based on the overlapping of the probability density functions (PDFs) between GCMs simulated and observed data for precipitation, maximum and minimum temperature^9^. Several studies were carried out for different climate variables using skill score indicator^2,6,7,9,10^. In addition, other performance indicators used were Nash Sutcliffe efficiency^2,4^, normalized root mean square error/deviation, absolute mean bias error/deviation, correlation coefficient^3,5,10,11^. Another commonly used and an important method of ranking of GCMs is the multicriteria decision-making (MCDM). It is a dynamic method for multicriteria ranking of alternatives by selecting the best one from several alternatives. In most of the MCDM methods, quantitative weights are assigned for different indicators to assess the relative importance of different indicators^21–25^. Different MCDM methods result in different outcomes in ranking GCMs^26^ and hence, the compromise solution approach is crucial in the MCDM method. The basis of the compromise solution is a concept of feasible solution which is closest to the ideal one^27^. VlseKriterijuska Optimizacija I Komoromisno Resenje (VIKOR)^28^ is a compromise solution method adopted by various researchers in material selection^29,30^, performance evaluation^24,31–33^, sustainable and renewable energy^34–37^ and water resources planning^38,39^.

From the literatures, it is evident that there are only a few studies exploring the utility of MCDM for ranking of GCMs. Also, it was observed that the ranking of GCMs related to the latest version of coupled model inter comparison project i.e., CMIP6, are limited for India. To the best of authors’ knowledge, no study of ranking CMIP6 GCMs using compromise solution method namely, VIKOR has been done yet. Hence in this study, the ranking of GCMs from CMIP6 for projected changes in temperature over India was carried out with 30 GCMs using VIKOR method. Both the maximum and minimum temperatures were used to perform critical assessment of GCMs from CMIP6. Understanding these temperatures can help to predict the climate extremes, weather patterns and make informed decisions. Maximum temperature is important for studies related to extreme weather events such as urban heat islands (UHIs) and hot spell^40^. At the same time, the minimum temperature is needed for determining nocturnal UHI and agriculture related studies^41,42^. Five performance indicators, namely absolute normalized mean bias error (ANMBE), correlation coefficient (CC), normalized root mean square error (NRMSE), Nash–Sutcliffe efficiency (NSE), and Perkins’s skill score (PSS), are considered in this study. The entropy method was adopted to assign weights to various indicators, and best-ranked GCMs were computed using VIKOR method.

### Study area and data collection

The Indian subcontinent lying in the northern hemisphere, with longitude and latitude ranging from 67.5° to 97.5° E and 7.5° to 37.5° N, respectively covering 335 numbers of one-degree spatial resolution grids, was considered as the study area. The model-simulated data from World Climate Research Programme (WCRP) was utilized to acquire GCMs under CMIP6 (https://esgf-node.llnl.gov/search/cmip6/) as a part of IPCC’s sixth assessment report^1^. Outputs from 30 GCMs for maximum and minimum temperature (designated as TMAX and TMIN) with daily temporal resolution were used as historical simulated data. The details of 30 GCMs under CMIP6 used in this study are tabulated in Supplementary Table S1. The gridded daily TMAX and TMIN data (https://www.imdpune.gov.in/lrfindex.php) for 50 years (1965–2014) at 1˚ spatial resolution are collected from the Indian Meteorological Department (IMD). These data are used as the historical observed data to evaluate the performance of climate models. The base period 1965–2014 was selected considering CMIP6 historical simulation data sets' availability until 2014. Also, CMIP6 is an updated version of CMIP5 to produce relatively higher resolution data with an increased number of distinct climate models and eight future scenarios representing shared-socioeconomic pathways. All the gridded GCMs data available at different spatial resolutions were brought down to a common grid resolution of 1° × 1° using bilinear interpolation techniques^3,43,44^.

It was observed from the previous studies that model selection should be done rationally for climate change impact studies^4,10,11,20,45^. Each CMIP6 model differs from each other, and within each model, different ensemble members result in different GCM outputs. Considering these facts, 30 GCMs were selected for the analysis such that all the models belong to the same modeling structure (i.e., Atmospheric General Circulation Model) and with the same ensemble realizations r1i1p1f1 (indicating realization index, initialization index, physics index, and forcing index as 1).

## Methodology

### Selection and evaluation of performance indicators

Performance indicators are statistical metrics used for testing the capability of GCMs in simulating the observed data (IMD gridded daily temperature data). No universally agreed criteria exists for selecting performance indicators for GCM assessment^20^. Hence, performance indicators are chosen from different categories and distributed into two scenarios. Scenario 1 (S1) has absolute normalized mean bias error (ANMBE), correlation coefficient (CC), normalized root mean square error (NRMSE), and Perkins’s skill score (PSS), whereas scenario 2 (S2) has an additional indicator Nash–Sutcliffe efficiency (NSE) apart from S1. These indicators were selected to ensure that at least one belongs to each category of error, correlation, and skill score. Mathematical representation of above discussed indicators is shown in supplementary equations S1 to S5.

### Normalization and weight computation of performance indicators

Normalization methods were adopted to measure various non-proportional performance indicators on a common scale^21,22,31,46–48^. In this study, the normalization is carried out using the *Max–Min* method to obtain the decision matrix. After normalization, the equity contribution for each indicator is calculated using the *Sum* method. Using the contributing values, the weights are computed using the Entropy method. The lower entropy value of the indicator corresponds to its more valuable information, i.e., larger entropy-based weight. Finally, the weighted normalized decision matrix is calculated which will subsequently be used as an input in MCDM method. Mathematical representation of above discussed normalization steps is shown in supplementary equations S6 to S10.

### Multicriteria Decision-Making using VIKOR

**Figure 1:**
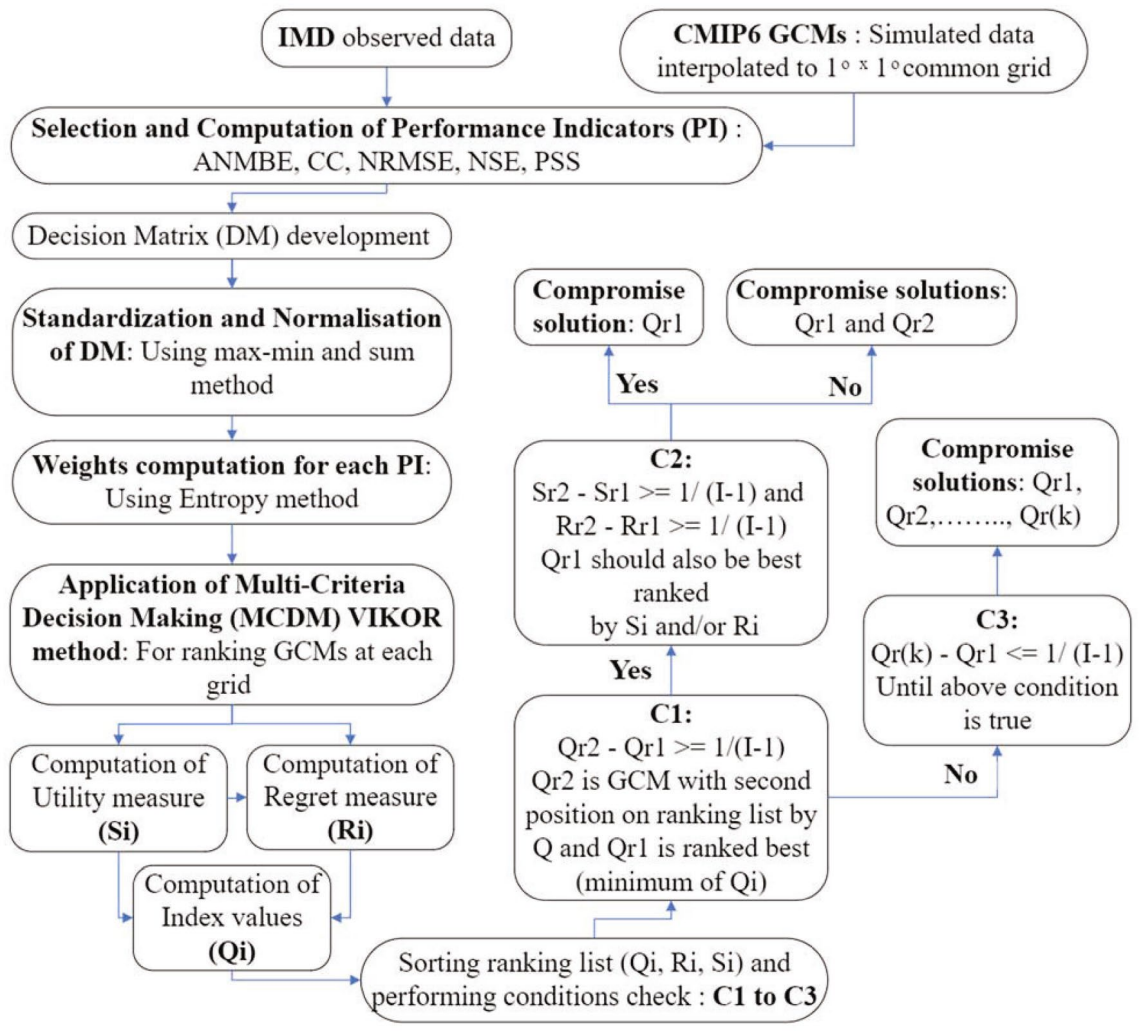
Methodology for ranking GCMs to obtain compromise solution using VIKOR method.

VIKOR (VlseKriterijuska Optimizacija I Komoromisno Resenje), primarily developed by Opricovnic in 1979, is a well-known multicriteria decision-making (MCDM) method^49^. VIKOR, a compromise ranking method, yields the feasible solution nearest to the ideal, hence helping the decision makers to conclude final solutions^39,47^. The methodology for ranking GCMs to obtain compromise solution using this method is described in Fig. 1. The computation of utility measure (*S*_*i*_), regret measure (*R*_*i*_) and the index values (*Q*_*i*_) are carried out using the following equations:

1 $$S_{i} = \mathop \sum \limits_{j = 1}^{J} \frac{{\left( {P_{ij} } \right)_{max} - P_{ij} }}{{\left( {P_{ij} } \right)_{max} - \left( {P_{ij} } \right)_{min} }}$$

2 $$R_{i} = \mathop {max }\limits_{j} \left\{ {\frac{{\left( {P_{ij} } \right)_{max} - P_{ij} }}{{\left( {P_{ij} } \right)_{max} - \left( {P_{ij} } \right)_{min} }}} \right\}$$

3 $$Q_{i} = \vartheta \left\{ {\frac{{S_{i} - \mathop {min}\limits_{i} \left( {S_{i} } \right)}}{{\mathop {max}\limits_{i} \left( {S_{i} } \right) - \mathop {min}\limits_{i} \left( {S_{i} } \right)}}} \right\} + \left( {1 - \vartheta } \right)\left\{ {\frac{{R_{i} - \mathop {min}\limits_{i} \left( {R_{i} } \right)}}{{\mathop {max}\limits_{i} \left( {R_{i} } \right) - \mathop {min}\limits_{i} \left( {R_{i} } \right)}}} \right\}$$

Here, $\vartheta$ is a balancing factor between the utility measure (overall benefit) and the regret measure (maximum individual deviation). The value of $\vartheta$ ranges between 0 and 1, with “*Voting by majority rule*” ($\vartheta$ > 0.5) or “*by consensus*” (for $\vartheta$ = 0.5) or “*with a veto*” (for $\vartheta$ < 0.5)^29,30,47,48^.

### Group decision-making method

The study area consists of 335 grids, each with a distinctive rank. In order to create a combined rank for the study area, a group decision-making process^25^ was adopted. The steps involved in this method are as follows:

At each grid, rankings were first separated into two halves and organized in descending order. GCMs with ranking 1 to X make up the first half of the sample (X = *I*/2, where *I* is the total number of GCMs). The GCM $i$ strength is stated as follows:

4 $$S_{i} = \mathop \sum \limits_{k = 1}^{a} \mathop \sum \limits_{z}^{x} \left( {x - z + 1} \right) q_{iz}^{k} \forall i, \;\;k \forall z = 1, \ldots ,x$$

where, $q_{iz}^{k}$ = 1 if GCM $i$ is in rank *z* for the grid point *k* and zero in all other case. $i$ corresponds to the GCM in the first half portion and *z* ranges from 1st to *x*th rank, and *k* represents the grid points.

The weakness of GCM $i$ is given as:

5 $$W_{i} = \mathop \sum \limits_{k = 1}^{a} \mathop \sum \limits_{z = y}^{I} \left( {z - y + 1} \right) q_{iz}^{k} \forall i,\;\; k \forall z = y, \ldots ,I$$

where, $q_{iz}^{k}$ = 1 if GCM $i$ is in rank *z* for the grid point *k* and zero in all other case. $i$ corresponds to the GCM in the second half portion and *z* ranges from 1st to *y*th rank up to last ranking in the portion, and *k* represents the grid points.

Net strength is calculated as:

6 $$N_{i} = S_{i} - W_{i}$$

The GCM with the highest net strength was regarded as the most appropriate or the best, and others were ordered in accordance with their values.

### Ethical approval

Ethics violation has not been done in the study.

## Results and discussions

Due to the complex atmospheric processes, model structure, and parameterization variability in representing land surface processes (vegetation dynamics, soil moisture, and land–atmosphere interactions), the temperature data may be over or underestimated from different CMIP models. Variable numerical schemes, grid configurations, spatial grid resolutions in capturing small-scale features, and representation of climate forcing using datasets and methods (for aerosols, land-use changes, greenhouse gas concentrations, ocean circulations, ice and snow albedo, and aerosols) contribute to differences among GCMs, even for same realization, initialization, physics, and forcings^20,50–52^. Therefore, there is a need to appraise the uncertainties associated with climate change data before incorporating it in hydro-climatological studies. Hence, the analysis was carried out for the entire Indian sub-continent consisting of 335 grids. But for demonstrating the performance evaluation of various GCMs for TMAX and TMIN, a grid with a longitude 94.5° E and a latitude 26.5° N located in North-East India was selected. The detailed description of the behavior of different GCMs on different performance indicators are explained in the following sub section.

### Performance evaluation of GCMs based on correlation Coefficient for grid (94.5° E, 26.5° N)

**Figure 2:**
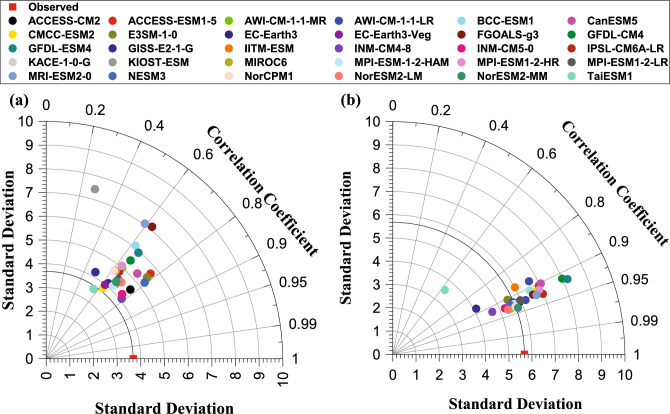
Taylor Diagram for (**a**) TMAX and (**b**) TMIN of 30 CMIP6 GCMs and the IMD gridded data.

Figure 2a and b depict the Taylor Diagram^53^ showing the correlation between the observed and the simulated temperature data from 30 GCMs for TMAX and TMIN, respectively. It can be observed from Fig. 2a (for TMAX data) that the GCMs, NESM3, INM-CM4-8, E3SM-1-0, ACCESS-ESM1-5, ACCESS-CM2, and INM-CM5-0 are having the highest CC values of 0.7919, 0.7843, 0.7798, 0.7766, 0.7742, and 0.7632, respectively. While KACE-1-0-G and KIOST-ESM are the worst correlated to the observed data with a CC value of − 0.0548 and 0.2774, respectively. Most of the GCM for TMAX (25 in number) had CC values between 0.6 and 0.8, exhibiting moderate matching with the observed data. Similarly, from Fig. 2b, it can be observed that the CC values of 25 models falls between 0.90 and 0.95 for TMIN. Hence, most of the models performed well in simulating the observed minimum temperature for the demonstration grid compared to TMAX. The KACE-1-0-G was the only GCM with small negative correlation for TMIN and TMAX, and hence not shown in Fig. 2. It is not prudent to ascertain the best GCMs for TMAX and TMIN only based on CC. Therefore, the following section further evaluates the GCMs based on other performance indicators before ascertaining the best GCMs for TMAX and TMIN.

### Analysis of performance indicators, entropy and VIKOR method at a grid (94.5° E, 26.5° N)

For demonstrating entropy and the VIKOR method, minimum temperature at a grid with a longitude 94.5° E and a latitude 26.5° N located in North-East India was selected. The analysis used the performance indicators under two scenarios, S1: ANMBE-CC-NRMSE-PSS and S2: ANMBE-CC-NRMSE-NSE-PSS and the results are listed in Table 1. From Table 1, it can be observed that the GCM, NESM3 is having the maximum similarity, PSS (97.02%) with the observed PDF and is the most preferred GCM. Other performance indicators also suggest that the same GCM performs better with the values of ANMBE (0.0218), NRMSE (0.1118), and NSE (0.8697). Similarly, INM-CM4-8 showed the least similarity (51.79%) with the observed PDF and was least preferred. Also, BCC-ESM1 was the least preferred in the case of indicators ANMBE (0.4965), NRMSE (0.5183), and NSE (− 1.8007). NorESM2-MM was the best correlated to observed data with a value of 0.9379, while KACE-1-0-G was the worst correlated to the observed data with a value of − 0.1273. The above analysis reveals that the indicators behave differently with distinct GCMs.

**Table 1:** Performance indicator (PI) values, utility measure (S), regret measure (R), and index values (Q) for minimum temperature, for each GCM under both scenarios S1 and S2, for the grid (94.5° E, 26.5° N) in North-East India.

Model name/PI	ANMBE	CC	NRMSE	NSE	PSS	S (S1)	R (S1)	Q (S1)	S (S2)	R (S2)	Q (S2)
ACCESS-CM2	0.1263	0.9249	0.1681	0.7055	0.8653	1.8836	0.8616	0.6938	2.8221	0.9385	0.8319
ACCESS-ESM1-5	0.1203	0.9055	0.1900	0.6236	0.8493	1.8916	0.8076	0.6424	2.7994	0.9078	0.7869
AWI-CM-1-1-MR	0.1149	0.9222	0.1792	0.6655	0.8508	1.9160	0.8343	0.6792	2.8395	0.9235	0.8165
AWI-ESM-1-1-LR	0.2088	0.9262	0.2417	0.3913	0.7014	1.8991	0.6806	0.5170	2.7200	0.8208	0.6559
BCC-ESM1	0.4965	0.9071	0.5183	− 1.8007	0.5191	1.0473	0.9973	0.4973	1.0473	0.9973	0.4966
CanESM5	0.3482	0.9026	0.3848	− 0.5435	0.6003	1.5044	0.8179	0.4983	1.9751	0.8179	0.4794
CMCC-ESM2	0.0908	0.9161	0.1646	0.7175	0.8837	1.9369	0.8701	0.7236	2.8799	0.9430	0.8511
E3SM-1-0	0.0221	0.9036	0.1303	0.8231	0.9616	2.0183	0.9994	0.8869	3.0009	0.9994	0.9521
EC-Earth3	0.1737	0.9187	0.2085	0.5467	0.8170	1.8021	0.7621	0.5607	2.6811	0.8791	0.7222
EC-Earth3-Veg	0.1612	0.9244	0.1954	0.6020	0.8389	1.8052	0.7943	0.5945	2.7049	0.8997	0.7545
FGOALS-g3	0.2731	0.9208	0.3017	0.0513	0.6291	1.7712	0.7542	0.5404	2.4647	0.7542	0.5105
GFDL-CM4	0.2730	0.9137	0.3339	− 0.1619	0.7416	1.4622	0.5054	0.1656	2.0759	0.6137	0.2385
GFDL-ESM4	0.1858	0.9192	0.2722	0.2278	0.7853	1.6971	0.6544	0.4100	2.4567	0.7596	0.5156
GISS-E2-1-G	0.1218	0.8779	0.1927	0.6128	0.6806	2.3002	0.8009	0.7988	3.2040	0.9038	0.8755
IITM-ESM	0.4238	0.8776	0.4452	− 1.0657	0.5180	1.3810	1.0000	0.6332	1.6563	1.0000	0.6412
INM-CM4-8	0.2743	0.9207	0.2999	0.0627	0.5179	2.0317	1.0000	0.8928	2.7295	1.0000	0.8900
INM-CM5-0	0.3044	0.9257	0.3241	− 0.0948	0.5180	1.8996	0.9999	0.8400	2.5384	0.9999	0.8455
IPSL-CM6A-LR	0.2181	0.9282	0.2614	0.2877	0.7751	1.6676	0.6320	0.3755	2.4496	0.7820	0.5430
KACE-1-0-G	0.0638	− 0.1273	0.4759	− 1.3605	0.9455	2.0706	1.0000	0.9084	2.2354	1.0000	0.7754
KIOST-ESM	0.3025	0.8822	0.3414	− 0.2149	0.5731	1.7813	0.8780	0.6696	2.3752	0.8780	0.6499
MIROC6	0.1261	0.9325	0.1609	0.7300	0.8728	1.8749	0.8791	0.7081	2.8226	0.9477	0.8439
MPI-ESM-1-2-HAM	0.2018	0.9235	0.2294	0.4515	0.7148	1.9074	0.7108	0.5508	2.7508	0.8434	0.6922
MPI-ESM1-2-HR	0.1369	0.9155	0.1949	0.6041	0.8448	1.8445	0.7956	0.6115	2.7450	0.9005	0.7648
MPI-ESM1-2-LR	0.1844	0.9208	0.2165	0.5115	0.7928	1.8044	0.7425	0.5418	2.6703	0.8658	0.7026
MRI-ESM2-0	0.0906	0.9251	0.1611	0.7294	0.8818	1.9445	0.8787	0.7354	2.8920	0.9474	0.8596
NESM3	0.0218	0.9224	0.1118	0.8697	0.9702	2.0140	1.0000	0.8858	3.0140	1.0000	0.9559
NorCPM1	0.2507	0.9326	0.2733	0.2211	0.6188	1.9087	0.7769	0.6182	2.6658	0.7769	0.5864
NorESM2-LM	0.2002	0.9332	0.2306	0.4459	0.7315	1.8820	0.7079	0.5378	2.7233	0.8413	0.6832
NorESM2-MM	0.1127	0.9379	0.1585	0.7382	0.9050	1.8513	0.8852	0.7047	2.8020	0.9507	0.8430
TaiESM1	0.3359	0.6299	0.4113	− 0.7632	0.5777	1.8637	0.8678	0.6922	2.2522	0.8678	0.6083
Entropy (S1)	0.9375	0.9892	0.9277		0.9259						
Degree of dispersion (S1)	0.0625	0.0108	0.0723		0.0741						
Normalized weights (S1)	0.2844	0.0493	0.3290		0.3373						
Entropy (S2)	0.9375	0.9892	0.9277	0.8767	0.9259						
Degree of dispersion (S2)	0.0625	0.0108	0.0723	0.1233	0.0741						
Normalized weights (S2)	0.1822	0.0316	0.2108	0.3593	0.2161						

Further, all the indicators were normalized using the Max–Min method and then the equity contribution for each indicator was calculated using the Sum method. Indicator values are made consistent with the requirements of the entropy approach by the normalization procedures, which also guarantee that large range indicators do not overpower the small range indicators. For S1, among the four indicators, PSS has the highest importance (33.73%) which shows that its effect on GCM ranking is the highest, followed by NRMSE (32.90%), ANMBE (28.44%) and CC (4.93%). There is no significant difference in the contribution of PSS, NRMSE, and ANMBE and their total contribution amount to 95.07%, making them an equally important indicator for GCM ranking for S1 at grid (94.5° E, 26.5° N). For S2, among the five indicators, NSE has the highest importance (35.93%), followed by PSS (21.61%), NRMSE (21.08%), ANMBE (18.22%), and CC (3.16%). The entropy method makes it easy to rank 30 GCMs by providing differential weights opportunities instead of equal weights. Weights computed by entropy methods were used to obtain a normalized weighted decision matrix, subsequently used as inputs to the VIKOR method. The compromise solution is obtained by computing utility measure (*S*_*i*_), regret measure (*R*_*i*_), and index values (*Q*_*i*_) using Eqs. (1–3). In this study, the balancing factor $\vartheta$ is taken as 0.5. GCM, GFDL-CM4 is identified as the compromise solution for both scenarios S1 and S2 by satisfying the conditions C1 and C2. From Table 1, it can be observed that GFDL-CM4 ranks the best by the measure Q (minimum value 0.1656 for S1 and 0.2385 for S2) and by measure R (minimum value of 0.5054 for S1 and 0.6137 for S2). The compromise solution was accepted as obtained by the minimum individual regret (minimum R value) of the “opponent”.

### Analysis of performance indicators, entropy and VIKOR method for India

The procedure described in previous section was repeated for the entire India comprising of 335 grids for minimum and maximum temperature using MATLAB and Python in-house developed code. For each grid, all the 30 GCMs were considered and 4 (or 5) indicators for scenario S1 (or S2) were used in achieving compromise solutions. It is noted that weights vary with indicators and with grids. The range of indicators depict a significant variation in the performance of various GCMs. A GCM may perform well in accordance with an indicator, and at the same time, the same GCM performs poorly in accordance with another indicator. One can refer the Supplementary Figs. S1–S10 for individual indicator values corresponding to all the 335 grids and all the GCMs over India, for TMAX and TMIN. For scenario S1, NRMSE is the most crucial indicator with a mean weightage of 41.18% and 45.88% for maximum and minimum temperatures, respectively. For scenario S2, NSE dominates NRMSE, with mean weightage of 35.30% and 42.95% for maximum and minimum temperature, respectively (Supplementary Fig. S11). Therefore, instead of assigning equal weights for indicators, differential weight opportunities were adopted for indicators using the entropy method.

The distribution of weights in various weight ranges (in %) obtained by the entropy method for the entire India, for scenarios S1 and S2 are listed in Table 2. It was observed that the number of grids with a weight less than 10% is the highest for CC (i.e., 323 for TMAX (S1), 318 for TMIN (S1), 333 for TMAX (S2), and 329 for TMIN (S2)), indicating that it is the least prominent indicator for ranking GCMs. Similarly, a greater number of grids in higher weight ranges indicate that NRMSE and NSE are the most prominent indicators for S1 and S2, respectively.

**Table 2:** Distribution of weights to performance indicators in various ranges under scenarios S1 and S2, over 335 grids of India.

Percentage weight range		(0,10]	(10,20]	(20,30]	(30,40]	(40,50]	(50,60]	(60,70]	(70,80]	(80,90]	(90,100]
TMAX (S1)	ANMBE	12	13	70	177	57	6	0	0	0	0
	CC	323	12	0	0	0	0	0	0	0	0
	NRMSE	5	11	23	63	198	35	0	0	0	0
	PSS	48	181	56	22	10	2	3	10	3	0
TMIN (S1)	ANMBE	3	14	30	133	149	6	0	0	0	0
	CC	318	14	2	1	0	0	0	0	0	0
	NRMSE	2	8	16	36	142	128	3	0	0	0
	PSS	253	31	12	16	10	6	4	2	1	0
TMAX (S2)	ANMBE	18	102	203	12	0	0	0	0	0	0
	CC	333	2	0	0	0	0	0	0	0	0
	NRMSE	6	15	282	32	0	0	0	0	0	0
	NSE	5	11	30	230	59	0	0	0	0	0
	PSS	171	112	25	10	1	4	9	3	0	0
TMIN (S2)	ANMBE	4	107	221	3	0	0	0	0	0	0
	CC	329	5	1	0	0	0	0	0	0	0
	NRMSE	4	19	303	9	0	0	0	0	0	0
	NSE	1	8	13	49	257	7	0	0	0	0
	PSS	274	21	21	8	5	3	2	1	0	0

**Figure 3:**
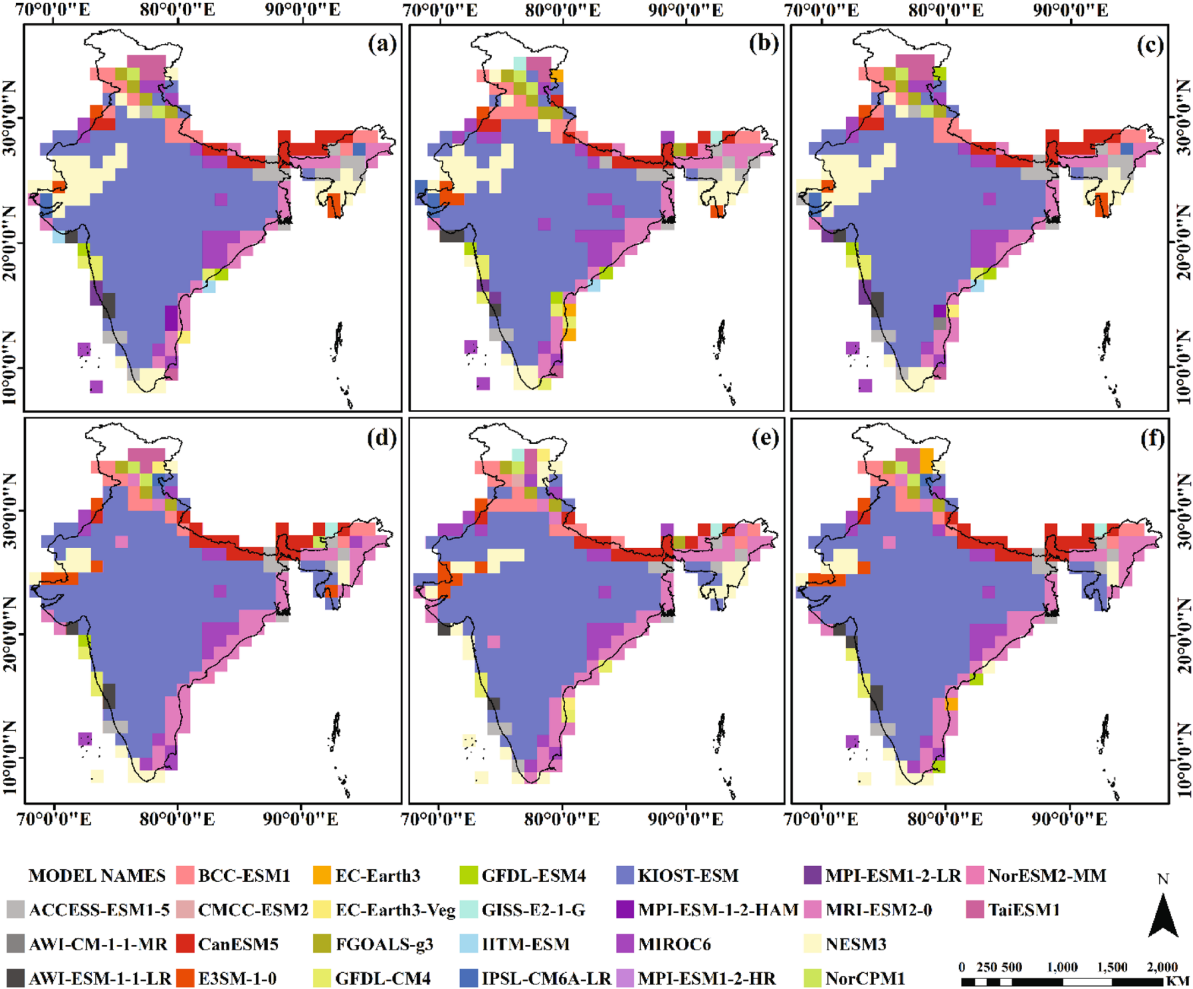
Spatial distributions of compromise solutions of maximum temperature for scenario S1 (a-c) and S2 (d-f)(Note: each figure is a complete compromise solution) (Maps created using ArcGIS Desktop 10.6.1, url: https://www.arcgis.com/index.html).

**Figure 4:**
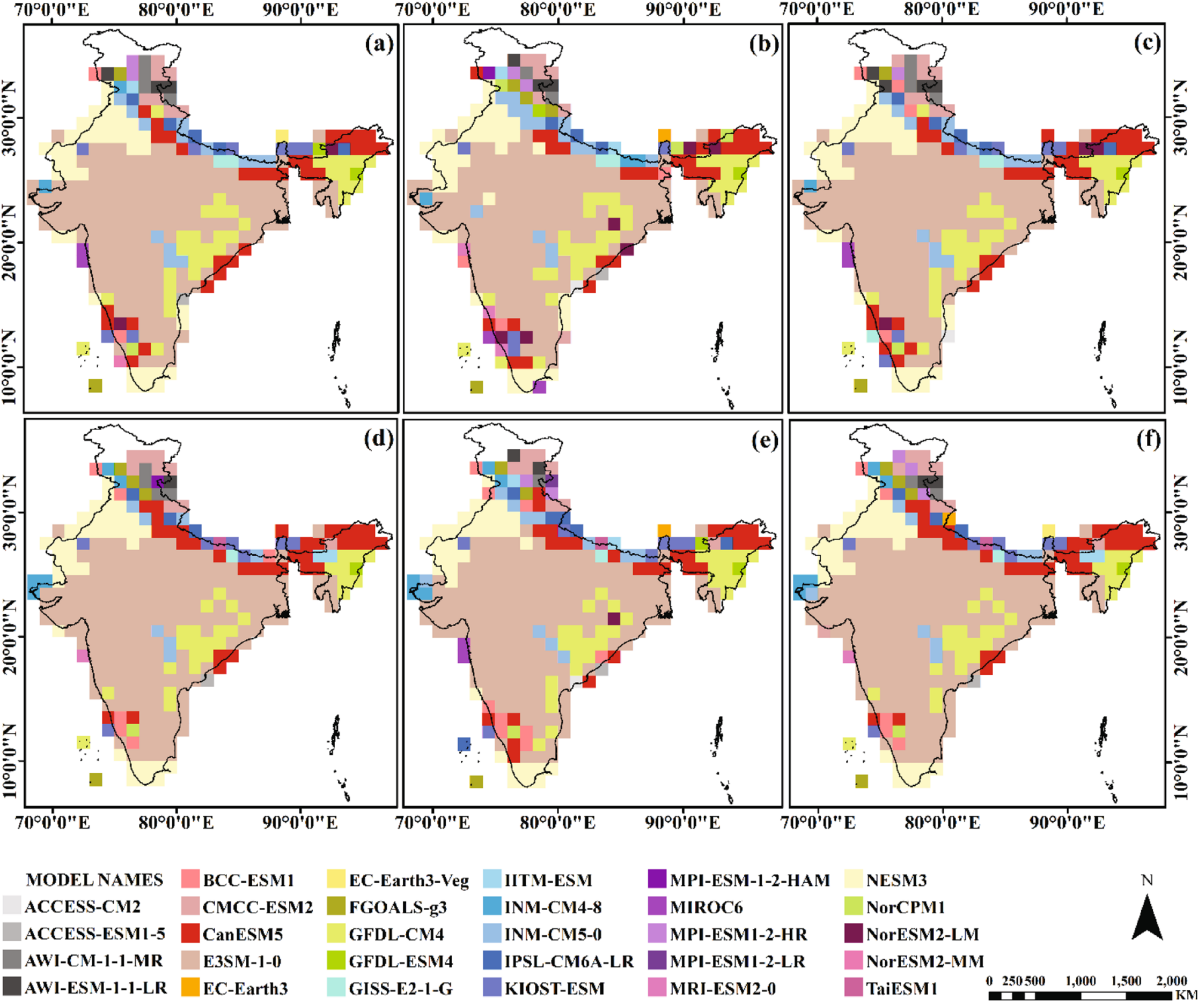
Spatial distributions of compromise solutions of minimum temperature for scenario S1 (a-c) and S2 (d-f) (Note: each figure is a complete compromise solution) (Maps created using ArcGIS Desktop 10.6.1, url: https://www.arcgis.com/index.html).

The compromise solutions were computed for entire India, and their solutions were accepted as obtained by the maximum group utility (minimum S value) of the “majority” and the minimum individual regret (minimum R value) of the “opponent”. From Figs. 3 and 4, it can be noticed that 301 grids (89.85%) for maximum temperature and 318 grids (94.92%) for minimum temperature, yield at least one same best-ranked GCMs for scenarios S1 and S2. By analyzing maximum and minimum temperatures from Figs. 3 and 4, it can also be observed that only 36 grids (10.75%) for S1 and 27 grids (8.06%) for S2, produced at least one same best-ranked GCMs. A uniform ranking pattern was seen in both scenarios as indicated by high similarity in compromise solutions (89.85% for maximum temperature and 94.92% for minimum temperature). Moreover, a nonuniform ranking pattern existed between maximum and minimum temperature under both scenarios (i.e., similarity under S1 was 10.75%, and S2 was 8.06%).

### Ensemble of GCMs identified for the Indian subcontinent

**Figure 5:**
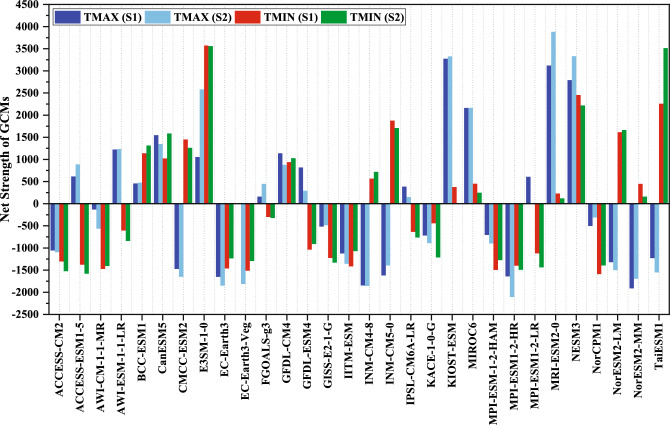
Net strength of GCMs under scenarios S1 and S2, for maximum and minimum temperatures.

It is evident that different grids have different best-ranked GCMs, and an effort was made to rank GCMs for the entire India. According to the group decision-making method discussed under methodology, the top five ranked GCMs for maximum temperature are KIOST-ESM, MRI-ESM2-0, NESM3, MIROC6, and CanESM5 for S1 and MRI-ESM2-0, NESM3, KIOST-ESM, E3SM-1-0, and MIROC6 for S2. The respective net strengths for the mentioned GCMs are 3275, 3121, 2791, 2162, and 1548 for S1 whereas 3883, 3331, 3329, 2583, and 2164 for S2 as shown in Fig. 5. The higher ranked GCMs are not mentioned as their net strength difference is more as compared to the top five mentioned GCMs. In the same way for minimum temperature, E3SM-1-0, NESM3, INM-CM5-0, CMCC-ESM2, CanESM5, and GFDL-CM4 are ranked first, second, fourth, sixth, eighth, and ninth for S1 and first, third, fourth, eighth, sixth, and ninth for S2 (listed in Table 3). The average ranking perspective (average of all ranks corresponding to each GCM over 335 grids) was also evaluated^54^. On the other hand, from average ranking method, it can be observed that MRI-ESM2-0, KIOST-ESM, NESM3, MIROC6 and CanESM5 are ranked first, second, third, fourth, and seventh for S1 and first, third, second, fifth and seventh for S2 (Table 3) for maximum temperature. Similarly, for minimum temperature, E3SM-1-0, NESM3, CMCC-ESM2, INM-CM5-0, and CanESM5 are ranked first, second, fourth, fifth, and eighth for S1 and first, third, sixth, fifth, and fourth for S2. For maximum temperature, MPI-ESM-1-2-HR, EC-Earth3, EC-Earth3-Veg, INM-CM4-8, NorESM2-MM, and EC-Earth3, EC-Earth3-Veg, KACE-1-0-G, NorESM2-LM, NorESM2-MM, are ranked among last five, under both scenarios S1 and S2, using group decision making and average ranking perspective, respectively. For minimum temperature, AWI-CM-1-1-MR, NorCPM1, EC-Earth3-Veg, MPI-ESM1-2-HR, ACCESS-ESM1-5 and EC-Earth3, EC-Earth3-Veg, KACE-1-0-G, ACCESS-ESM1-5, MPI-ESM-1-2-HAM, IITM-ESM, are ranked among last eights, under both scenarios S1 and S2, using group decision making and average ranking perspective, respectively.

**Table 3:** Combined ranks of GCMs for maximum and minimum temperatures, under both scenarios S1 and S2, using group decision-making and average perspective methods.

Model name	GDM ranks				Average perspective ranks			
	TMAX (S1)	TMAX (S2)	TMIN (S1)	TMIN (S2)	TMAX (S1)	TMAX (S2)	TMIN (S1)	TMIN (S2)
ACCESS-CM2	20	20	22	29	19	19	22	22
ACCESS-ESM1-5	10	8	23	30	12	9	29	29
AWI-CM-1-1-MR	15	17	27	26	15	16	20	23
AWI-ESM-1-1-LR	**6**	**7**	17	17	**6**	**6**	17	18
BCC-ESM1	12	10	**7**	**7**	16	14	**7**	**8**
CanESM5	**5**	**6**	**8**	**6**	**7**	**7**	**8**	**4**
CMCC-ESM2	24	25	**6**	**8**	24	26	**4**	**6**
E3SM-1-0	8	**4**	**1**	**1**	**5**	**4**	**1**	**1**
EC-Earth3	27	28	26	21	28	25	24	24
EC-Earth3-Veg	29	27	29	23	27	28	27	28
FGOALS-g3	14	11	15	15	14	13	15	15
GFDL-CM4	**7**	9	**9**	**9**	8	8	**12**	**9**
GFDL-ESM4	9	12	19	18	9	10	21	17
GISS-E2-1-G	17	16	21	24	18	18	26	21
IITM-ESM	21	21	25	19	20	20	28	27
INM-CM4-8	28	29	10	10	23	22	11	10
INM-CM5-0	25	22	**4**	**4**	25	24	**5**	**5**
IPSL-CM6A-LR	13	13	18	16	11	12	16	16
KACE-1-0-G	19	18	16	20	30	27	30	30
KIOST-ESM	**1**	**3**	13	14	**2**	**3**	13	11
MIROC6	**4**	**5**	11	11	**4**	**5**	10	14
MPI-ESM-1-2-HAM	18	19	28	22	17	17	25	26
MPI-ESM1-2-HR	26	30	24	28	21	21	19	20
MPI-ESM1-2-LR	11	14	20	27	10	11	18	19
MRI-ESM2-0	**2**	**1**	14	13	**1**	**1**	14	12
NESM3	**3**	**2**	**2**	**3**	**3**	**2**	**2**	**3**
NorCPM1	16	15	30	25	13	15	23	25
NorESM2-LM	23	23	**5**	**5**	26	29	**6**	**7**
NorESM2-MM	29	26	12	12	29	30	9	13
TaiESM1	22	24	**3**	**2**	22	23	**3**	**2**

Bold values represent the significant ranks of GCMs.

After the extensive analysis of compromise solutions for 335 grids individually, group decision-making, and average raking perspective as a group for both scenarios S1 and S2, it can be suggested that no single GCM is suitable for India as a whole. An ensemble of GCMs can be employed for climate change impact studies for temperature data. Further, the ensemble of GCMs has been recommended by considering India's average cumulative percentage coverage as listed in Table 4, group decision-making, and average ranking perspective. It was observed that KIOST-ESM covers an average 50.85% with compromise solution 1 (CS1) at 173 grids, CS2 at 165, and CS3 at 173 grids for scenario S1 for maximum temperature. Also, KIOST-ESM covers an average of 57.71% with CS1 at 196 grids, CS2 at 188, and CS3 at 196 grids for scenario S2 for maximum temperature. Similarly, E3SM-1-0 covers an average 50.15% for scenario S1 and 53.53% for S2 minimum temperature. Notably, percentage coverage has increased from scenarios S1 to S2 by including NSE as an additional indicator. The KIOST-ESM, MRI-ESM2-0, MIROC6, NESM3, and CanESM5, collectively cover 80.30% (S1) and 85.57% (S2) of India and can be used as an ensemble for maximum temperature. Furthermore, E3SM-1-0, NESM3, CanESM5, GFDL-CM4, INM-CM5-0, and CMCC-ESM2 collectively cover 87.26% (S1) and 87.16% (S2) of India and can be used as an ensemble for minimum temperature.

**Table 4:** Number of grids over which respective GCMs are compromise solutions (compromise solutions less than seven grids are not tabulated).

Model name	CS1	CS2	CS3	Average cumulative % coverage
	TMAX (S1)			
KIOST-ESM	173	165	173	50.85
NESM3	36	33	35	61.19
MRI-ESM2-0	26	30	25	69.25
MIROC6	20	24	20	75.62
CanESM5	16	15	16	80.30
ACCESS-ESM1-5	15	13	16	84.68
BCC-ESM1	14	14	14	88.86
	TMAX (S2)			
KIOST-ESM	196	188	196	57.71
MRI-ESM2-0	40	40	39	69.55
MIROC6	18	19	17	74.93
NESM3	15	25	16	80.50
CanESM5	18	14	19	85.57
BCC-ESM1	12	15	12	89.45
	TMIN (S1)			
E3SM-1-0	171	162	171	50.15
NESM3	39	41	39	61.99
GFDL-CM4	34	34	34	72.14
CanESM5	30	28	31	81.00
INM-CM5-0	13	15	13	85.07
CMCC-ESM2	7	7	8	87.26
	TMIN (S2)			
E3SM-1-0	183	173	182	53.53
NESM3	36	35	35	64.08
CanESM5	33	28	32	73.33
GFDL-CM4	28	31	28	81.99
INM-CM5-0	7	11	8	84.58
CMCC-ESM2	9	8	9	87.16

ACCESS-CM2, INM-CM4-8, INM-CM5-0, KACE-1-0-G, and MPI-ESM1-2-HR have never been compromise solutions for any of the grids, while AWI-CM-1-1-MR, CMCC-ESM2, EC-Earth3, EC-Earth3-Veg, GISS-E2-1-G, IITM-ESM, MPI-ESM-1-2-HAM, and NorESM2-MM were compromise solutions for three or fewer grids. Also, these GCMs were ranked among the last fifteens using group decision and average perspective methods and hence are the less prominent GCMs for maximum temperature. Moreover, KACE-1-0-G was not found to be suitable for any of the grids and ACCESS-CM2, ACCESS-ESM1-5, EC-Earth3, EC-Earth3-Veg, GFDL-ESM4, GISS-E2-1-G, IITM-ESM, MPI-ESM-1-2-HAM, MPI-ESM1-2-HR, MPI-ESM1-2-LR, and NorCPM1 to three or fewer grids, as compromise solutions for minimum temperature. Also, these GCMs were ranked among the last 15 using group decision and average perspective methods and hence are the less prominent GCMs for minimum temperature.

Ensembles of CNRM-CM5, FGOALS-s2, and MIROC5 for the maximum temperature and MIROC4h, NorESM1-M, MIROC5, and CESM1-CAM5 for the minimum temperature were already proposed in the literature^11^ using compromise programming and a group decision approach. It is found out that the GCMs ensemble for maximum temperature included MIROC5 and MIROC6, respectively, from this study as well as from the literature^11^. Both the GCMs are from the same modeling institution, the Atmosphere and Ocean Research Institute, University of Tokyo, Japan. Both studies produce distinct GCMs ensemble suggestions for maximum and minimum temperature, which might be a result of different chosen performance indicators, decision-making approaches, spatial resolutions, and model selections.

## Conclusions

This study deals with identifying the best ensemble of GCMs for the Indian subcontinent for studying the futuristic climate change impact. The identification was based on five performance indicators under two scenarios, S1 (ANMBE-CC-NRMSE-PSS) and S2 (ANMBE-CC-NRMSE-NSE-PSS), for ranking 30 CMIP6 GCMs. Grid wise performance was evaluated using these indicators at 335 grids for maximum and minimum temperature. The entropy method was operated to assign weights to the indicators, after normalizing their values using the max–min and the sum methods. Based on indicators and their assigned weights, a multicriteria decision-making method VIKOR was used to rank GCMs and obtain compromise solutions at all grids. Group decision-making, average ranking perspective and cumulative percentage coverage of India, collectively, were used to suggest an ensemble of GCMs. It is understood that seasonal changes and precipitation influences surface temperature. However, this study has not accounted seasonal influences for identifying the best GCMs. A detailed study is needed to understand the model biases associated with seasonal changes and precipitation. The conclusions based on the analysis from this study are summarized as follows:

1. For scenario S1, NRMSE is the most crucial indicator, with a mean weightage of 41.18% and 45.88% for maximum and minimum temperature, respectively. For scenario S2, NSE dominates NRMSE, with mean weightage of 35.30% and 42.95% for maximum and minimum temperature, respectively.
2. Number of grids with weight less than 10% is the highest for CC, indicating it as the least prominent indicator. More number of grids in higher weight ranges indicate NRMSE and NSE as the most prominent indicators for S1 and S2, respectively.
3. Weights vary with indicators and grids, and a particular GCM may perform well considering an indicator, while the same GCM performs poorly considering other indicators. So, it necessitates considering multiple criteria for GCM assessment.
4. A uniform ranking pattern was seen in both scenarios as there was 89.85% similarity in compromise solutions of maximum temperature for S1 and S2, whereas it was 94.92% for minimum temperature. A nonuniform ranking pattern was observed for maximum and minimum temperature under both scenarios (i.e., similarity under S1 was 10.75%, and S2 was 8.06%).
5. No single GCM was suitable for the Indian region as a whole, and hence an ensemble of best GCMs was suggested. Ensemble of KIOST-ESM, MRI-ESM2-0, MIROC6, NESM3, and CanESM5 for maximum temperature and E3SM-1-0, NESM3, CanESM5, GFDL-CM4, INM-CM5-0, and CMCC-ESM2 for minimum temperature was recommended based on this study.

### Supplementary Information

Supplementary Information.

## Data Availability

The gridded precipitation data used in this study are collected from the Indian Meteorological Department Pune (https://www.imdpune.gov.in/lrfindex.php).
