# Supplementary material for: Identifying and ranking of CMIP6-global climate models for projected changes in temperature over Indian subcontinent
Source: Sci Rep. 2024 Feb 6;14:3076. doi: 10.1038/s41598-024-52275-1 (PMC10847151; doi:10.1038/s41598-024-52275-1)
Supplement: Supplementary file 1 — Supplementary Information. [file 41598_2024_52275_MOESM1_ESM.docx]

**Identifying and Ranking of CMIP6-Global Climate Models for Projected Changes in Temperature Over Indian Subcontinent**

**Abdul Rahman^1^ and Sreeja Pekkat^2*^**

^1^Research Scholar, Department of Civil Engineering, Indian Institute of Technology Guwahati, Guwahati, Assam-781039, India, ORCID: 0009-0000-6925-5544; Email: r.abdul@iitg.ac.in

^2^Associate Professor, Department of Civil Engineering, Indian Institute of Technology Guwahati, Guwahati, Assam-781039, India, ORCID: 0000-0001-9166-5590; Email: [sreeja@iitg.ac.in](mailto:sreeja@iitg.ac.in)

(*corresponding author)

**Performance indicators**

 (S1)

 (S2)

 (S3)

 (S4)

$PSS = \frac{1}{n} \sum_{i=1}^{ci} min\left(f_{sm} , f_{ob}\right)$ (S5)

where ${x_{ob}}_{i}$ and ${x_{sm}}_{i}$ are observed and simulated data, $\sigma_{ob}$ and $\sigma_{sm}$ are the standard deviation of observed and simulated data, $n$is the total number of data (i.e., 50 years data, which is equal to 18250 number of data points), ${\boxed{x_{ob}}}_{i}$ and ${\boxed{x_{sm}}}_{i}$ are mean of observed and simulated data, $f_{ob}$ and $f_{sm}$ are frequency of data in the given class interval from observed and simulated data, and $ci$ is the class interval used to calculate the PDF of data. Also, the class interval of 5˚C based on the TMAX and TMIN range of data is selected for PSS calculation.

**Normalization and weight computation of performance indicators**

The normalization using the *Max-Min* method to obtain the decision matrix:

$u_{ij}=\left\{\begin{matrix} \frac{p_{ij}-p_{j}^{min}}{p_{j}^{max}-p_{j}^{min}} \left|j\in PA\right. \\ \frac{p_{j}^{max}-p_{ij}}{p_{j}^{max}-p_{j}^{min}} \left|j\in NA\right. \end{matrix}\right. and u_{ij}\in\left[0,1\right]$ (S6)

where, $p_{ij}$ is indicator values, *j* is the index of the indicators ( *j*=1,…, *J* where *J*=4 for S1 and *J*=5 for S2), *i* is the index of GCMs ( *i*=1,…., *I* where *I*=30), $u_{ij}$ max-min normalized value of indicator and *PA* and *NA* are positive and negative attributes^1,2^.

The equity contribution for each indicator using the *Sum* method:

**$v_{ij}=\frac{u_{ij}}{\sum_{i=1}^{I} u_{ij}} and \sum_{i=1}^{I} v_{ij}=1$** (S7)

where $v_{ij}$ is the contributing values of each model corresponding to each indicator.

The weights computation using the Entropy method:

**$\varepsilon_{j}=-\frac{1}{\ln\left(I\right)}\sum_{i=1}^{I} v_{ij} \ln\left(v_{ij}\right) and \varepsilon_{j}\in\left[0,1\right]$** (S8)**$w_{j}=\frac{1-\varepsilon_{j}}{J-\sum_{j=1}^{J} \varepsilon_{j}} and \sum_{j=1}^{J} w_{j}=1$**  (S9)

where, $\varepsilon_{j}$ is the entropy value of *j-th* indicator (if $u_{ij}$ = 0, then $v_{ij}$ ln ($v_{ij}$) = 0), $w_{j}$ is the weight of *j-th* indicator. The value (1-$\varepsilon_{j}$) is the degree of dispersion indicating the internal information of each indicator^3,4,5^.

The weighted normalized decision matrix using

**$P_{ij}=w_{j} v_{ij}$** (S10)

where, $P_{ij}$ is the weighted normalized values of decision matrix.

**Table S1**: Details of 30 GCMs under CMIP6 models considered in this study

| **Country** | **Modeling Institute** | **Model Name** | **Spatial resolution (Long ˟ Lat )** | **Notation** |
| --- | --- | --- | --- | --- |
| Australia | Commonwealth Scientific and Industrial Research Organization- Australian Research Council Centre of Excellence for Climate System Science | ACCESS-CM2 | 1.875 ˟ 1.25 | M1 |
|  | Commonwealth Scientific and Industrial Research Organization | ACCESS-ESM1-5 | 1.875 ˟ 1.25 | M2 |
| Canada | Canadian Centre for Climate Modelling and Analysis, Environment and Climate Change Canada | CanESM5 | 2.81 ˟ 2.79 | M6 |
| China | Beijing Climate Center, Beijing | BCC-ESM1 | 2.81 ˟ 2.79 | M5 |
|  | Chinese Academy of Sciences, Beijing | FGOALS-g3 | 2.00 ˟ 2.025 | M11 |
|  | Nanjing University of Information Science and Technology, Nanjing | NESM3 | 1.875 ˟ 1.86 | M26 |
| Europe | Consortium of various institutions from Spain, Italy, Denmark, Finland, Germany, Ireland, Portugal, Netherlands, Norway, UK, Belgium, and Sweden | EC-Earth3 | 0.703 ˟ 0.702 | M9 |
|  | Consortium of various institutions from Spain, Italy, Denmark, Finland, Germany, Ireland, Portugal, Netherlands, Norway, UK, Belgium, and Sweden | EC-Earth3-Veg | 0.703 ˟ 0.702 | M10 |
| France | Institut Pierre Simon Laplace, Paris | IPSL-CM6A-LR | 2.5 ˟ 1.267 | M18 |
| Germany | Alfred Wegener Institute, Helmholtz Centre for Polar and Marine Research | AWI-CM-1-1-MR | 0.9375 ˟ 0.935 | M3 |
|  | Alfred Wegener Institute, Helmholtz Centre for Polar and Marine Research | AWI-ESM-1-1-LR | 1.875 ˟ 1.865 | M4 |
|  | Max Planck Institute for Meteorology | MPI-ESM-1-2-HAM | 1.875 ˟ 1.865 | M22 |
|  | Max Planck Institute for Meteorology | MPI-ESM1-2-HR | 0.9375 ˟ 0.935 | M23 |
|  | Max Planck Institute for Meteorology | MPI-ESM1-2-LR | 1.875 ˟ 1.865 | M24 |
| India | Indian Institute of Tropical Meteorology, Pune | IITM-ESM | 1.875 ˟ 1.905 | M15 |
| Italy | Fondazione Centro Euro-Mediterraneo sui Cambiamenti Climatici (CMCC), Lecce | CMCC-ESM2 | 1.25 ˟ 0.942 | M7 |
| Japan | Japan Agency for Marine‐Earth Science and Technology, Atmosphere and Ocean Research Institute, National Institute for Environmental Studies, and RIKEN Center for Computational Science | MIROC6 | 1.406 ˟ 1.401 | M21 |
|  | Meteorological Research Institute, Tsukuba | MRI-ESM2-0 | 1.125 ˟ 1.1215 | M25 |
| Korea | National Institute of Meteorological Sciences (NIMS) and Korea Meteorological Administration (KMA) | KACE-1-0-G | 1.875 ˟ 1.25 | M19 |
|  | Korea Institute of Ocean Science and Technology, Busan | KIOST-ESM | 1.875 ˟ 1.895 | M20 |
| Norway | Norwegian Earth System Model Climate Modeling Consortium | NorCPM1 | 2.5 ˟ 1.895 | M27 |
|  | Norwegian Earth System Model Climate Modeling Consortium | NorESM2-LM | 2.5 ˟ 1.895 | M28 |
|  | Norwegian Earth System Model Climate Modeling Consortium | NorESM2-MM | 1.25 ˟ 0.942 | M29 |
| Russia | Institute for Numerical Mathematics, Russian Academy of Science, Moscow | INM-CM4-8 | 2.0 ˟ 1.5 | M16 |
|  | Institute for Numerical Mathematics, Russian Academy of Science, Moscow | INM-CM5-0 | 2.0 ˟ 1.5 | M17 |
| Taiwan | Taiwan Earth System Model | TaiESM1 | 1.25 ˟ 0.942 | M30 |
| USA | Lawrence Livermore National Laboratory, Livermore | E3SM-1-0 | 1.0 ˟ 1.0 | M8 |
|  | National Oceanic and Atmospheric Administration, GFDL, Princeton | GFDL-CM4 | 1.25 ˟ 1.0 | M12 |
|  | National Oceanic and Atmospheric Administration, GFDL, Princeton | GFDL-ESM4 | 1.25 ˟ 1.0 | M13 |
|  | NASA Goddard Institute for Space Studies, New York | GISS-E2-1-G | 2.5 ˟ 2.0 | M14 |


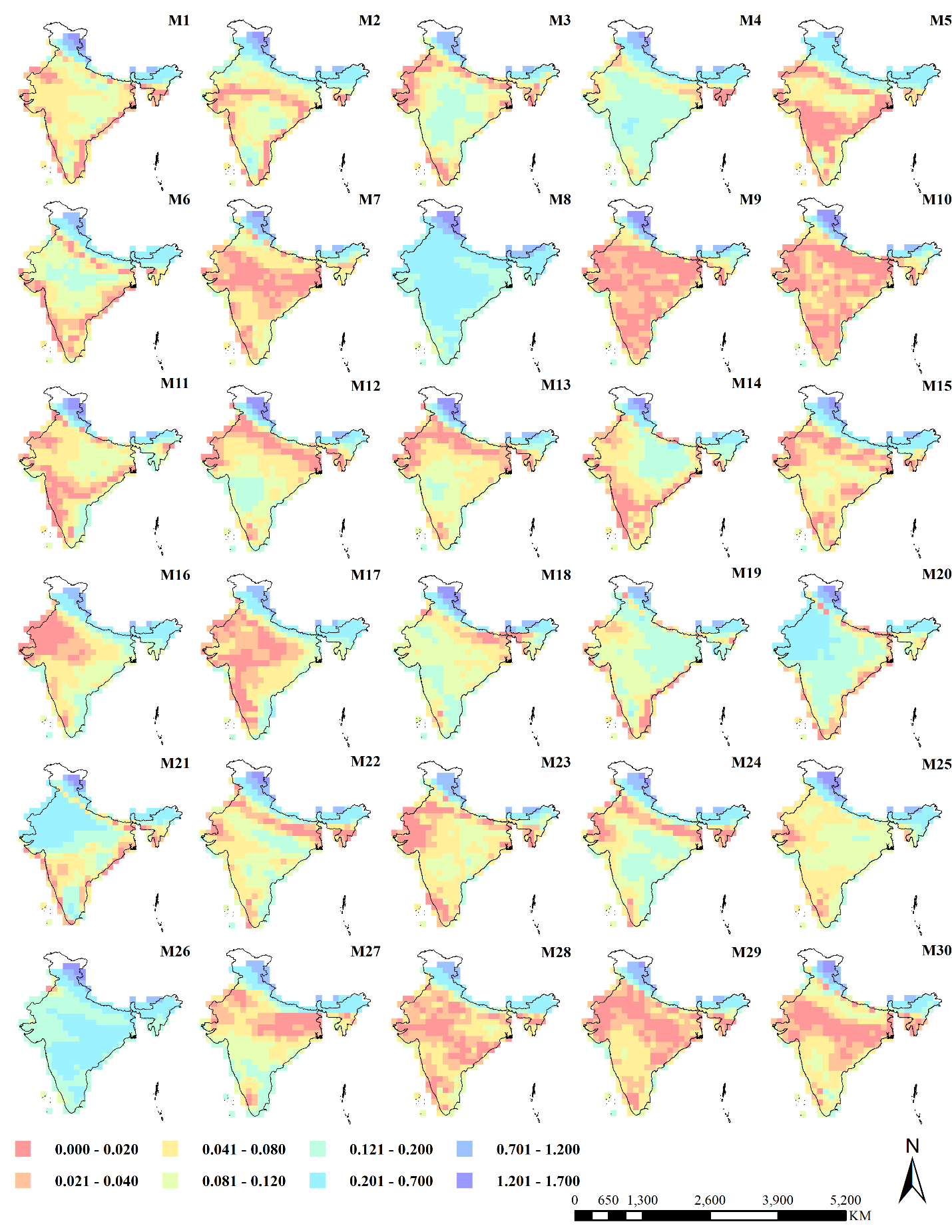
**Figure S1:** Absolute normalized mean bias error (ANMBE) performance indicator values for India, involving all 30 CMIP6 GCMs, for maximum temperature (Maps created using ArcGIS Desktop 10.6.1, url: https://www.arcgis.com/index.html)


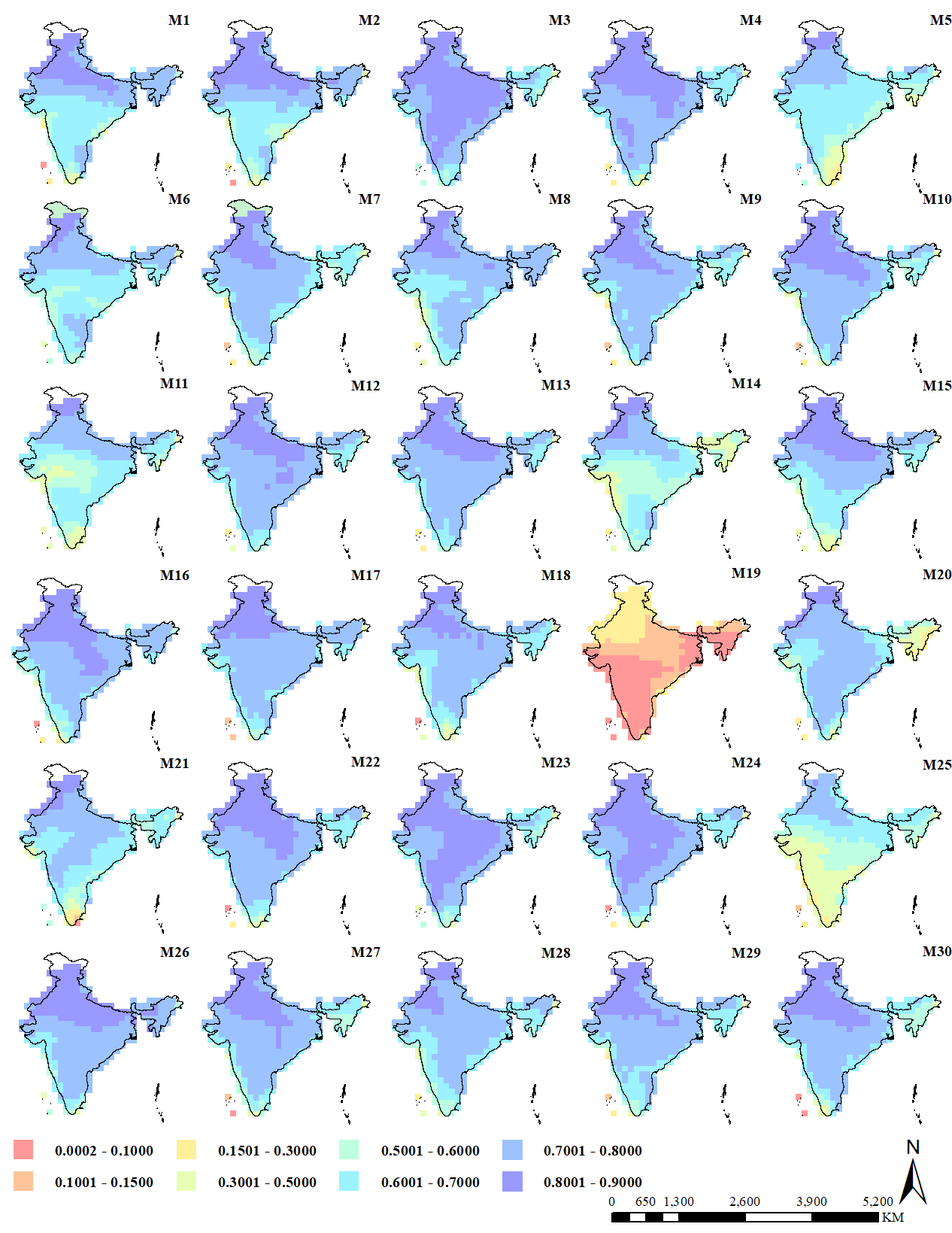


**Figure S2:** Correlation coefficient (CC) performance indicator values for India, involving all 30 CMIP6 GCMs, for maximum temperature (Maps created using ArcGIS Desktop 10.6.1, url: https://www.arcgis.com/index.html)


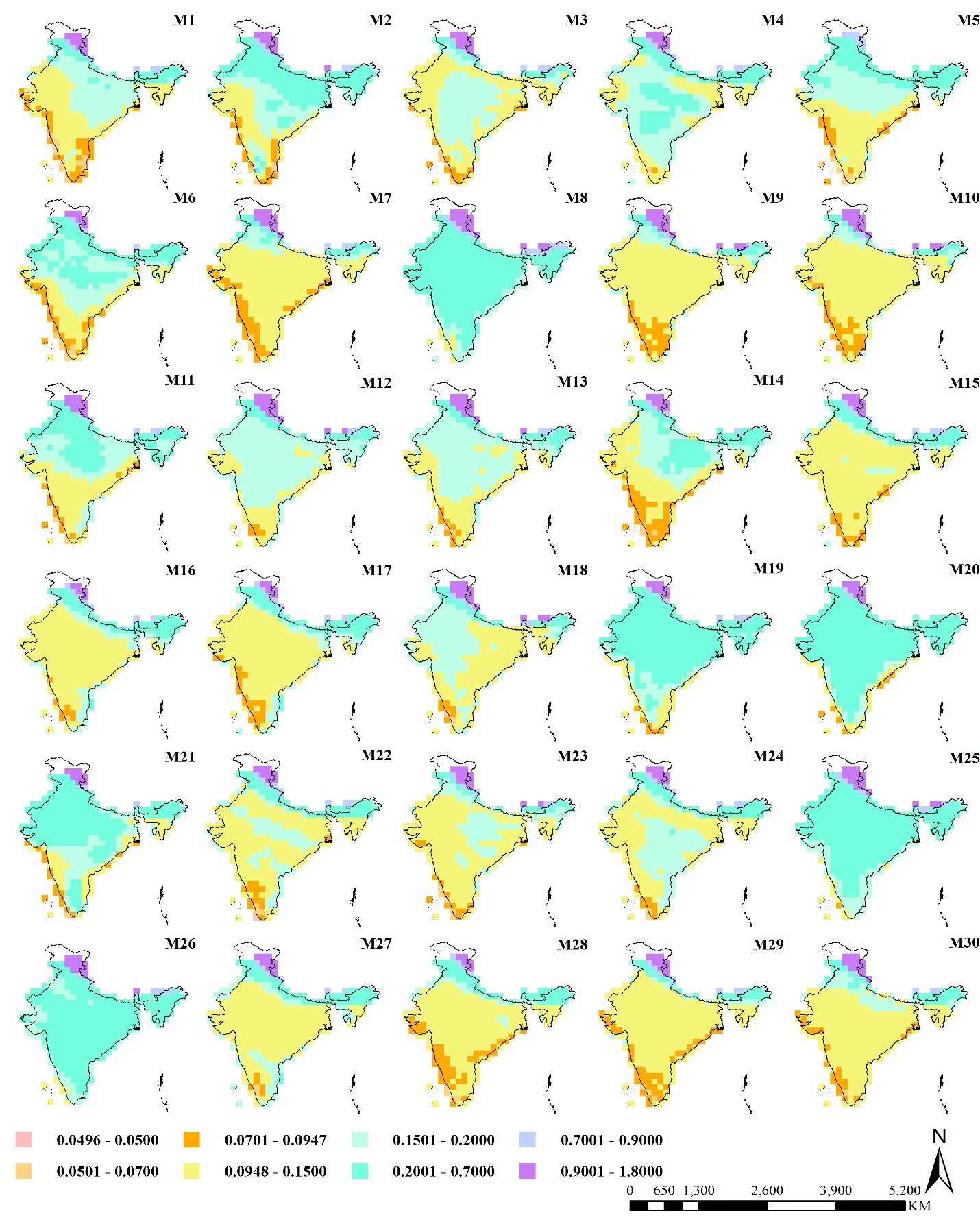


**Figure S3:** Normalized root mean square error (NRMSE) performance indicator values for India, involving all 30 CMIP6 GCMs, for maximum temperature (Maps created using ArcGIS Desktop 10.6.1, url: https://www.arcgis.com/index.html)


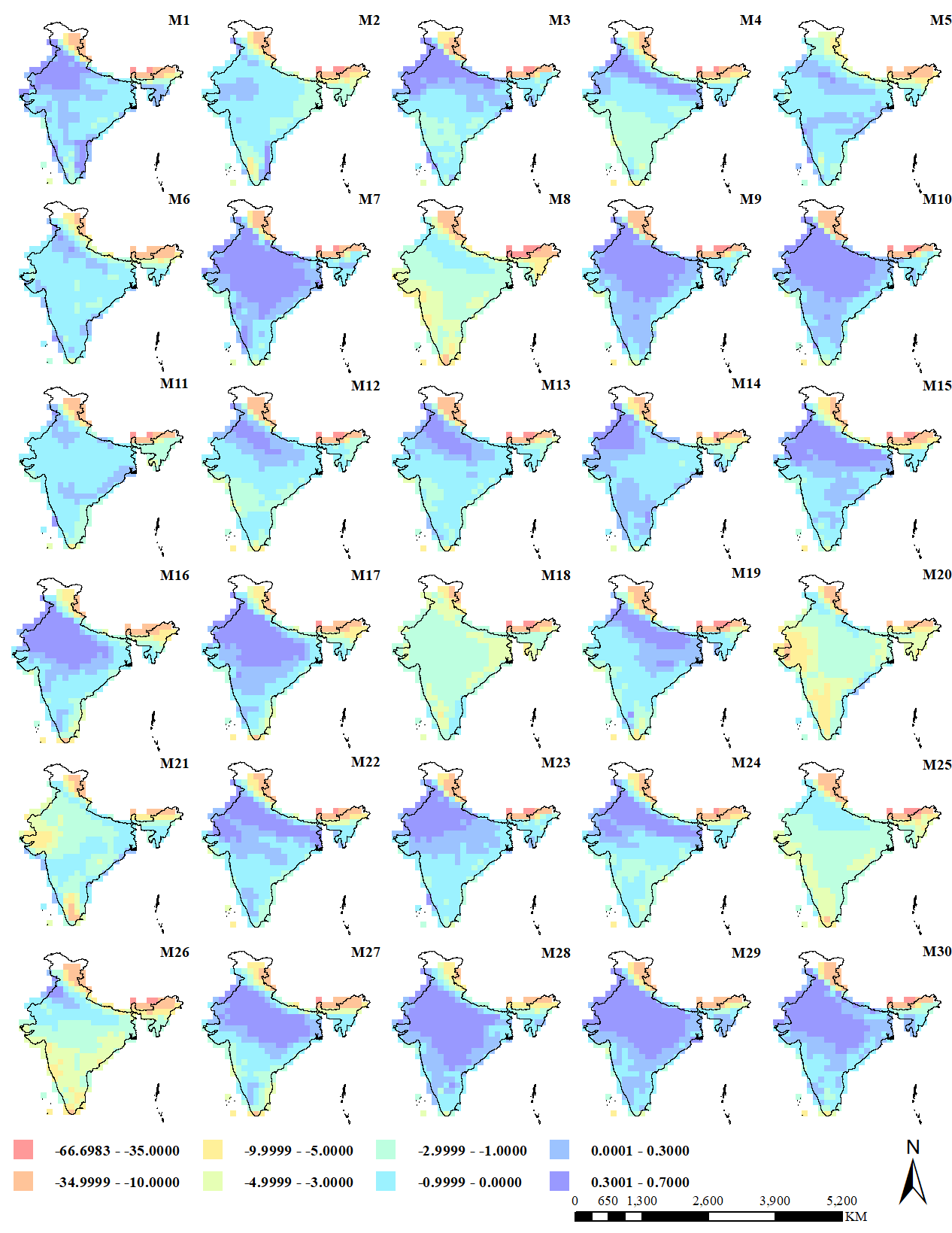


**Figure S4:** Nash-Sutcliffe efficiency (NSE) performance indicator values for India, involving all 30 CMIP6 GCMs, for maximum temperature (Maps created using ArcGIS Desktop 10.6.1, url: https://www.arcgis.com/index.html)


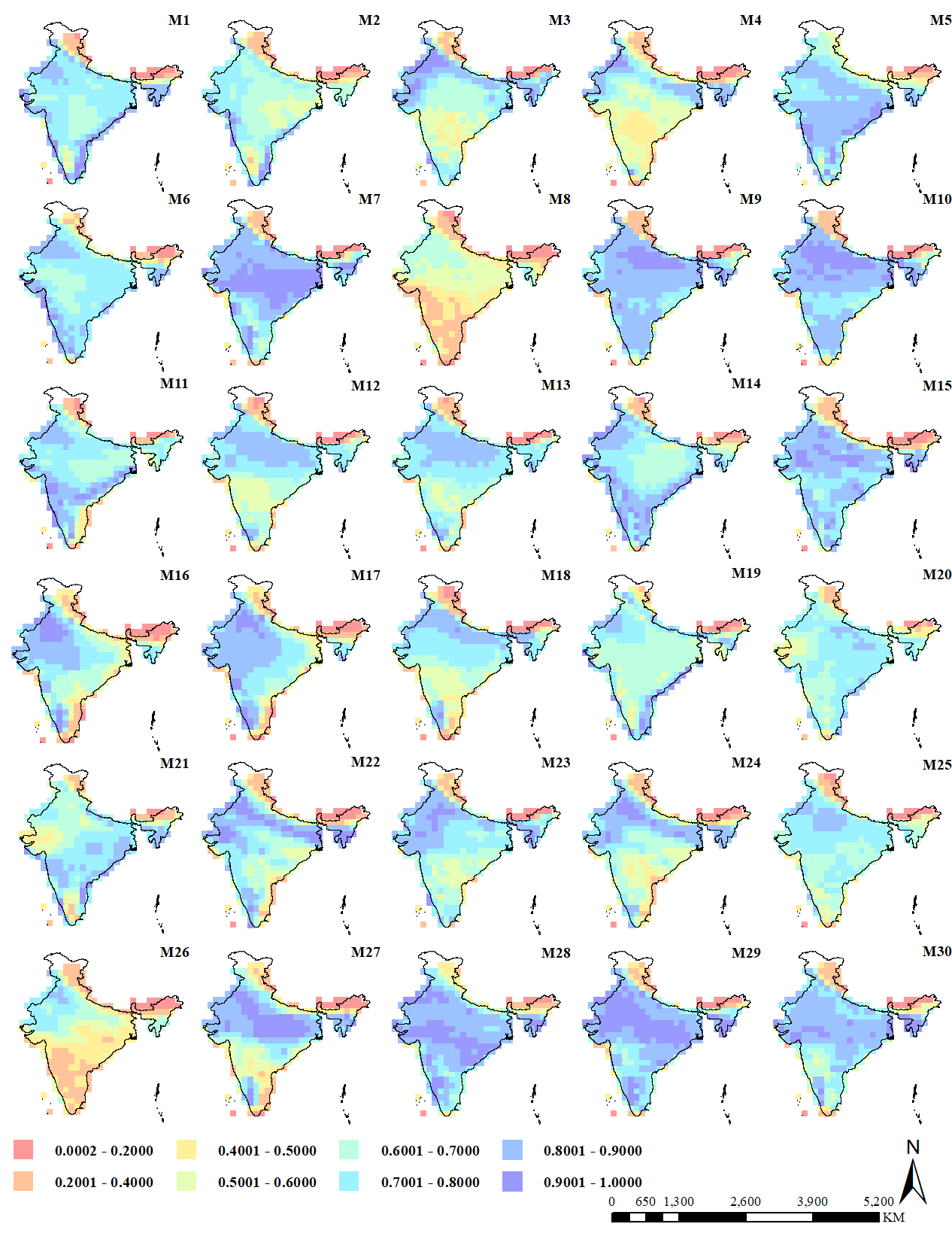


**Figure S5:** Perkins skill score (PSS) performance indicator values for India, involving all 30 CMIP6 GCMs, for maximum temperature (Maps created using ArcGIS Desktop 10.6.1, url: <https://www.arcgis.com/index.html>)


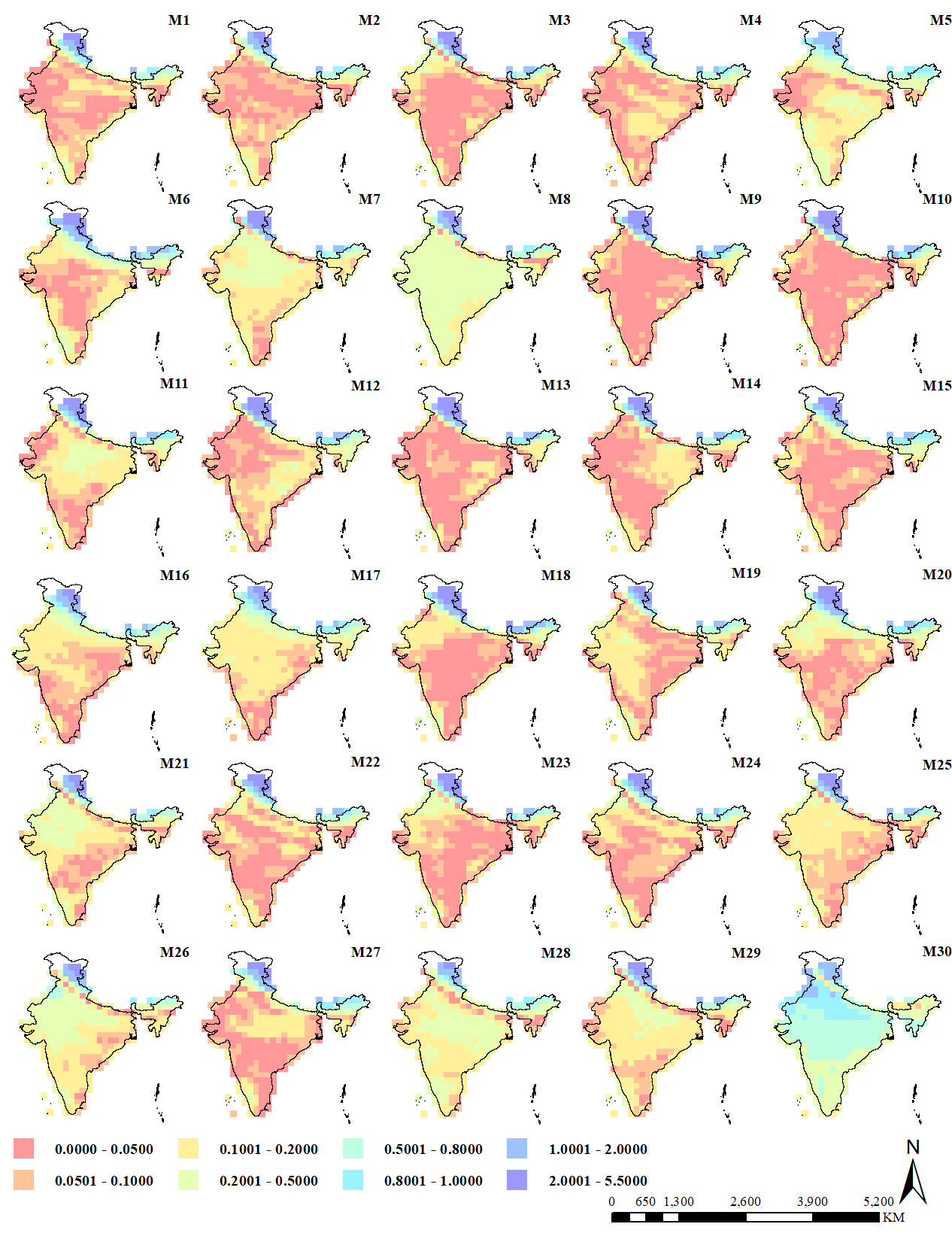


**Figure S6:** Absolute normalized mean bias error (ANMBE) performance indicator values for India, involving all 30 CMIP6 GCMs, for minimum temperature (Maps created using ArcGIS Desktop 10.6.1, url: https://www.arcgis.com/index.html)


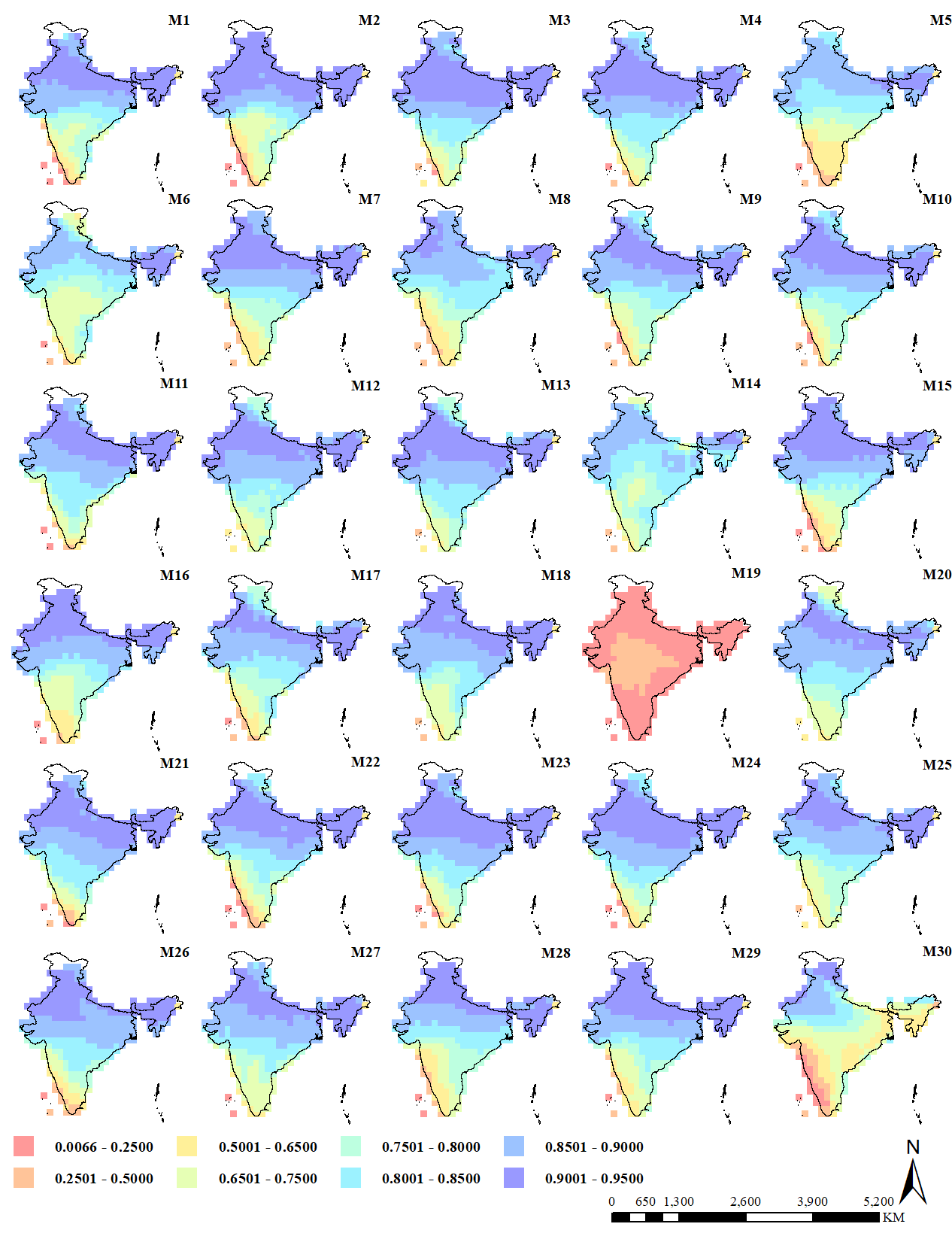


**Figure S7:** Correlation coefficient (CC) performance indicator values for India, involving all 30 CMIP6 GCMs, for minimum temperature (Maps created using ArcGIS Desktop 10.6.1, url: https://www.arcgis.com/index.html)


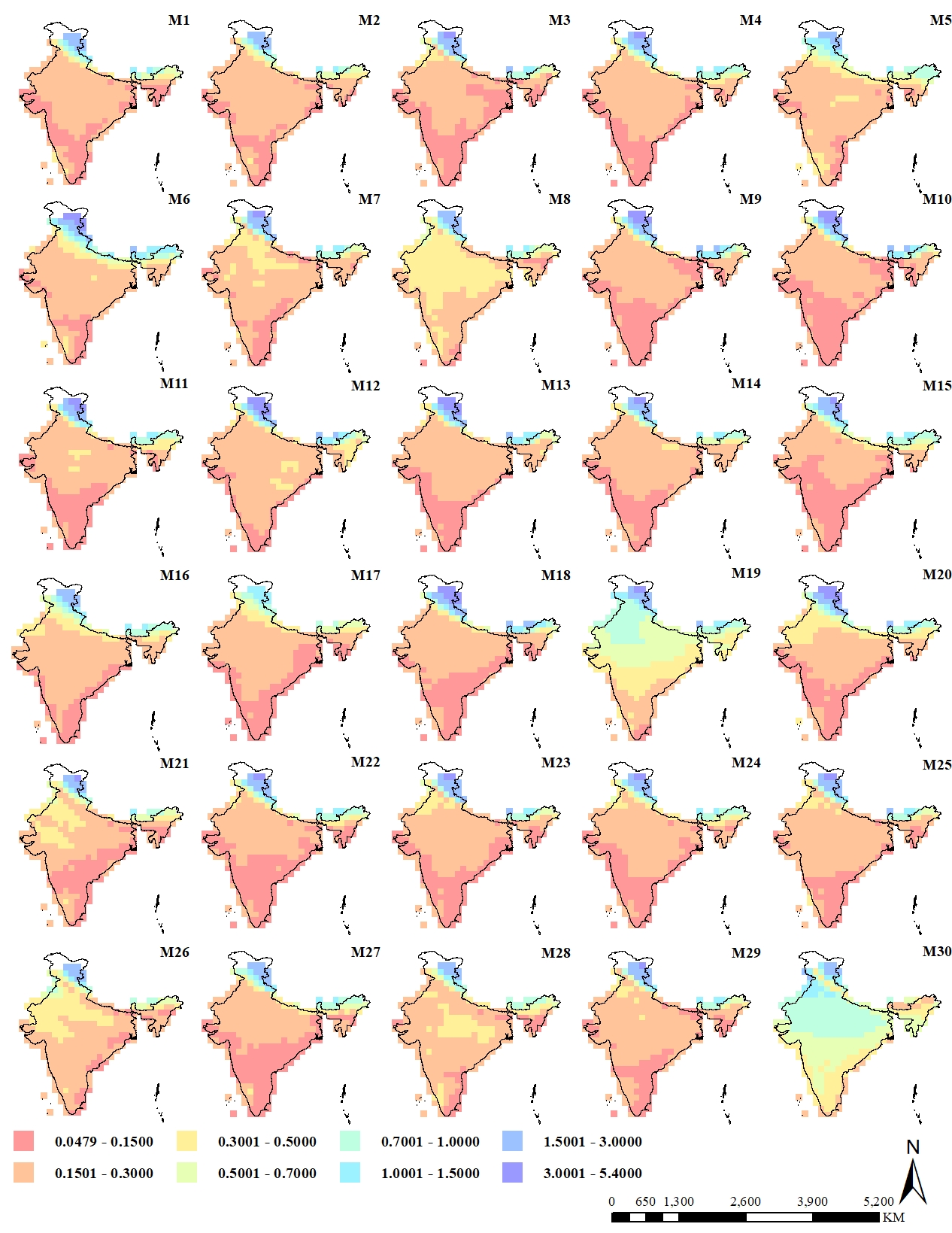


**Figure S8:** Normalized root mean square error (NRMSE) performance indicator values for India, involving all 30 CMIP6 GCMs, for minimum temperature (Maps created using ArcGIS Desktop 10.6.1, url: https://www.arcgis.com/index.html)


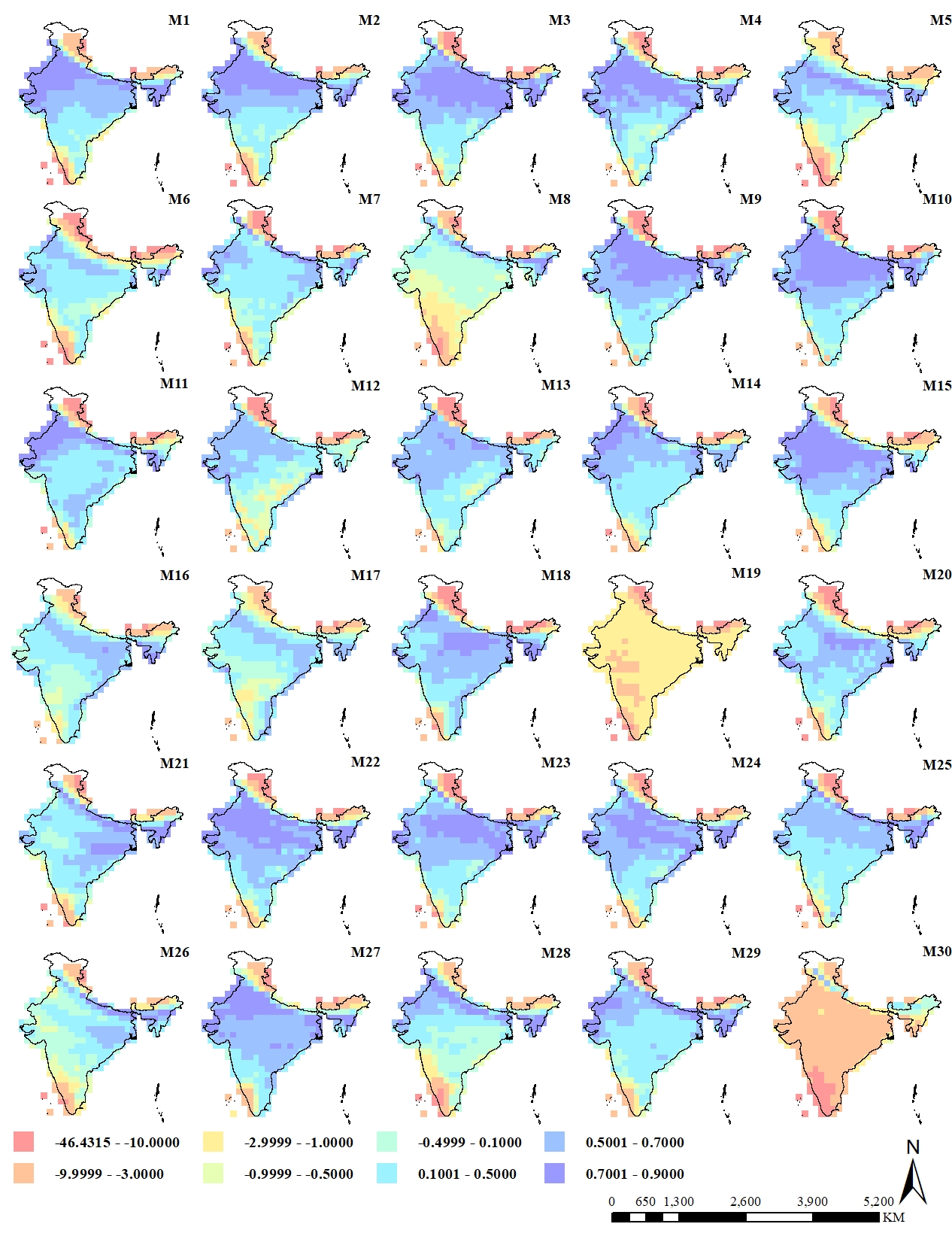


**Figure S9:** Nash-Sutcliffe efficiency (NSE) performance indicator values for India, involving all 30 CMIP6 GCMs, for minimum temperature (Maps created using ArcGIS Desktop 10.6.1, url: <https://www.arcgis.com/index.html>)


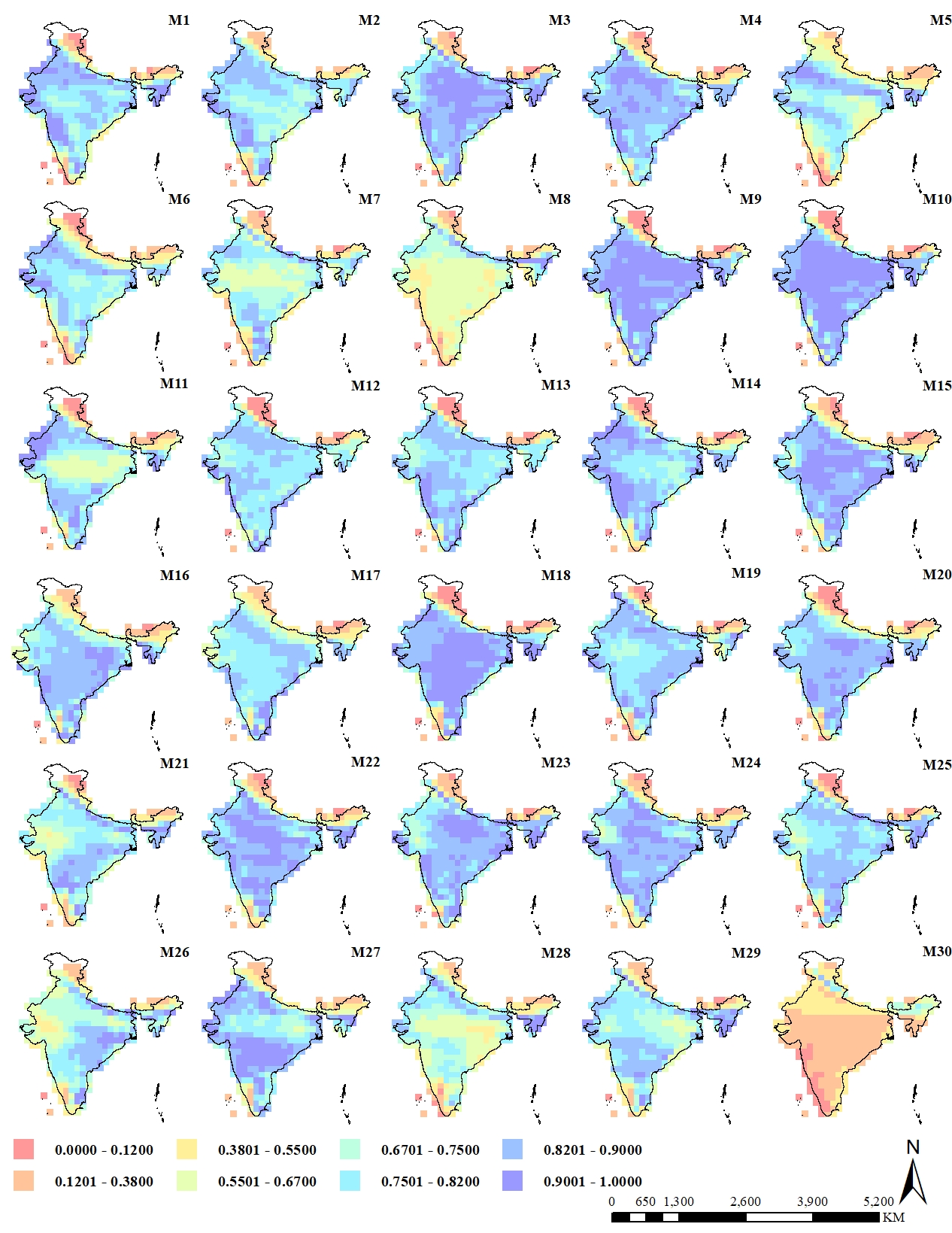


**Figure S10:** Perkins skill score (PSS) performance indicator values for India, involving all 30 CMIP6 GCMs, for minimum temperature (Maps created using ArcGIS Desktop 10.6.1, url: <https://www.arcgis.com/index.html>)





**Figure S11.** Spread of weights of performance indicators for maximum and minimum temperature under scenarios S1 (a and c) and S2 (b and d)

Note: Bars represent mean, plus and minus whisker of error bars as maximum and minimum weights of indicators, respectively.

Figure S11 presents performance indicators with bars representing means and error lines as maximum and minimum indicator weights. It was observed that for maximum temperature, ANMBE ranges from 5.69 to 54.69% (4.94 to 35.29%), CC ranges from 1.74 to 19.18% (1.52 to 17.54%), NRMSE ranges from 6.82 to 58.50% (6.27 to 33.62%), PSS ranges from 4.94 to 84.66% (3.22 to 77.79%) and NSE ranges from (8.11 to 44.69%), for scenarios S1 (S2). Similarly, for minimum temperature, ANMBE ranges from 5.79 to 53.76% (5.33 to 32.58%), CC ranges from 2.02 to 30.60% (1.34 to 21.05%), NRMSE ranges from 6.16 to 63.12% (5.66 to 31.42%), PSS ranges from 2.91 to 86.03% (1.60 to 79.15%) and NSE ranges from (7.99 to 52.31%), for scenario S1 (S2).

**References**

1. Jahan, A., Mustapha, F., Ismail, M. Y., Sapuan, S. M. & Bahraminasab, M. A comprehensive VIKOR method for material selection. *Mater Des* **32**, 1215–1221 (2011).
2. Zeng, Q. L., Li, D. D. & Yang, Y. Bin. VIKOR method with enhanced accuracy for multiple criteria decision making in healthcare management. *J Med Syst* **37**, (2013).
3. Mukhametzyanov, I. Z. Specific character of objective methods for determining weights of criteria in MCDM problems: Entropy, CRITIC, SD. *Decision Making: Applications in Management and Engineering* **4**, 76–105 (2021).
4. Li, X. *et al.* Application of the entropy weight and TOPSIS method in safety evaluation of coal mines. in *Procedia Engineering* vol. 26 2085–2091 (2011).
5. Pomerol, J.-C. & Barba-Romero, S. *Multicriterion Decision in Management*. vol. 25 (Springer US, 2000).
